# A Randomised Feasibility Study Assessing Acute Physiological Responses to Weight Stigma in Women Living With Obesity

**DOI:** 10.1111/cob.70073

**Published:** 2026-02-15

**Authors:** Adrian Brown, Jed Wingrove, Stuart W. Flint

**Affiliations:** ^1^ Centre for Obesity Research University College London London UK; ^2^ Bariatric Centre for Weight Management and Metabolic Surgery University College London Hospital NHS Trust London UK; ^3^ National Institute of Health Research UCLH Biomedical Research Centre London UK; ^4^ School of Psychology University of Leeds Leeds UK; ^5^ Scaled Insights, Nexus University of Leeds Leeds UK

**Keywords:** appetite, cortisol, experimental, obesity, physiological, stress, weight stigma

## Abstract

Experimental evidence indicates that exposure to weight stigma can lead to sustained elevations in cortisol and ambulatory blood pressure. However, little is known about its acute effects on other physiological markers. This study aimed to explore the feasibility of measuring the physiological response of weight stigma, a novel weight stigmatising paradigm and preliminary efficacy signals. In a prospective randomised feasibility study, women living with obesity were assigned to either a weight stigmatising or non‐weight stigmatising paradigm (15 min) and physiological response measured from baseline to 120 min. Eighteen women living with obesity were recruited (nine participants in each group; mean age 43.2 years (SD 10.3); body mass index of 45.8 kg/m^2^ (SD 5.9); 72.2% White ethnicity). Preliminary efficacy showed an observed acute increase in plasma cortisol (26.7 pg/mL, 95% CI −5.5 to 58.9), alongside increased systolic blood pressure (12.7 mmHg, 95% CI 0.6–24.8), self‐reported stress (17.4 mm 95% CI 1.6–33.3), appetite (hunger/desire to eat; 16.8 mm, 95% CI 2.0–31.7; 20.9 mm 95% CI 5.1–36.7, respectively), reduction in peptide YY (−11.8 pg/mL 95% CI −21.6 to −2.01) and fullness (−13.9 95% CI −27.6 to −0.3). No differences were found between the intervention and control group in all measured parameters. Furthermore, this study showed that recruitment, randomisation and measuring real‐time physiological response was feasible. This is the first feasibility study attempting to comprehensively characterise the acute physiological impact of experiencing weight stigma in people living with obesity in an experimental setting. Future appropriately powered studies are needed to confirm the preliminary efficacy findings.

## 1 Introduction

Weight stigma and discrimination have increased and are pervasive throughout society [1, 2]. UK data highlight that 88% of patients with obesity have reported experiences of weight stigma [3]. The common societal message that weight, and therefore obesity, is solely within an individual's control and thus is one's personal responsibility negates the scientific evidence regarding its complex aetiology. This reinforces negative stereotypes of people living with obesity such as laziness, gluttonous, and lacking willpower [1]. Weight stigma affects both women and men [4]. However, women typically report stronger and more frequent experiences of weight stigma than men [4, 5, 6, 7, 8], which may partly reflect the cultural emphasis on a thin ideal more commonly associated with women's bodies. To date there is limited research examining the physiological response of experiencing weight stigma [9, 10, 11].

Weight stigma has been shown to have detrimental effects on both mental and physical health, as well as health‐related behaviours [12]. Empirical evidence has shown weight stigma results in healthcare avoidance, emotional eating, greater energy intake and a greater likelihood of developing obesity [1]. However, the evidence linking the physiological impact of weight stigma is largely cross‐sectional, describing changes in blood pressure (BP), glycaemic control, stress cytokines, oxidative stress and mortality [13, 14].

Of the limited experimental studies investigating the physiological effect of exposure to weight stigma in people living with obesity, the focus has only examined the impact of salivary cortisol reactivity and BP [15, 16, 17]. These have shown weight stigma resulted in sustained cortisol elevation and negatively impacted ambulatory BP [17]. By only measuring a single outcome, this currently provides a limited understanding of the acute physiological response of experiencing weight stigma; therefore, a more holistic and comprehensive assessment is required.

The mechanisms underlying the negative physiological response to weight stigma experiences are not fully understood but may reflect those of chronic social stress [18]. Stress activates the hypothalamic–pituitary–adrenal (HPA) axis, immune and sympathetic systems [16], with chronic stress linked to numerous negative health outcomes [19]. Chronic activation of the HPA axis and elevated cortisol can result in tissue damage and fat storage [20] potentially contributing to obesity and disease development. The physiological response to experiences of other discriminatory behaviour such as racism has shown negative effects on BP [21, 22], cortisol and psychological stress [23, 24, 25].

Further scientific examination of the physiological impact of weight stigma experiences on people living with obesity is warranted to advance current understanding of this understudied area, to improve support provided to people living with obesity, and to inform weight stigma and discrimination interventions. Although some proponents have suggested that there is a causal pathway between experiences of weight stigma and negative health outcomes such as Type 2 diabetes and heart disease [18], evidence to date demonstrates an association with these disease states [26] rather than a causal relationship.

Therefore, this feasibility study aimed to evaluate the feasibility of recruitment, randomisation, assessment procedures and implementation of the intervention with the aim to facilitate the planning and the conduct of a full‐scale randomised controlled trial. It also aimed to explore preliminary efficacy signals and undertake preliminary characterisation of the physiological response to a novel weight stigma experience in female adults living with obesity.

## 2 Methods

### 2.1 Design and Ethical Approval

The study design was a prospective two‐arm randomised feasibility study conducted at University London Hospital (UCLH) in London, United Kingdom (UK). This study was conducted at the Clinical Research Facility at UCLH in accordance with the Helsinki Declaration of 1975 and with ethical approval by the Dulwich Research Ethics Committee (20/PR/0916). The study was registered at ClinicalTrials.gov under number NCT05001633.

### 2.2 Modified Aims of the Project

Both the participant information sheet (PIS) and the informed consent form had a modified project title ‘A pilot study to assess the impact of exposure to popular media content on patients living with obesity’ and within the PIS, the aim of the study was not fully disclosed. This was done to prevent an anticipatory stress response and to avoid misleading the control participants about the content of the media clips and speaking task. The study aims were fully explained to study participants at the end of the study visit as part of the debriefing session.

### 2.3 Recruitment

**FIGURE 1:**
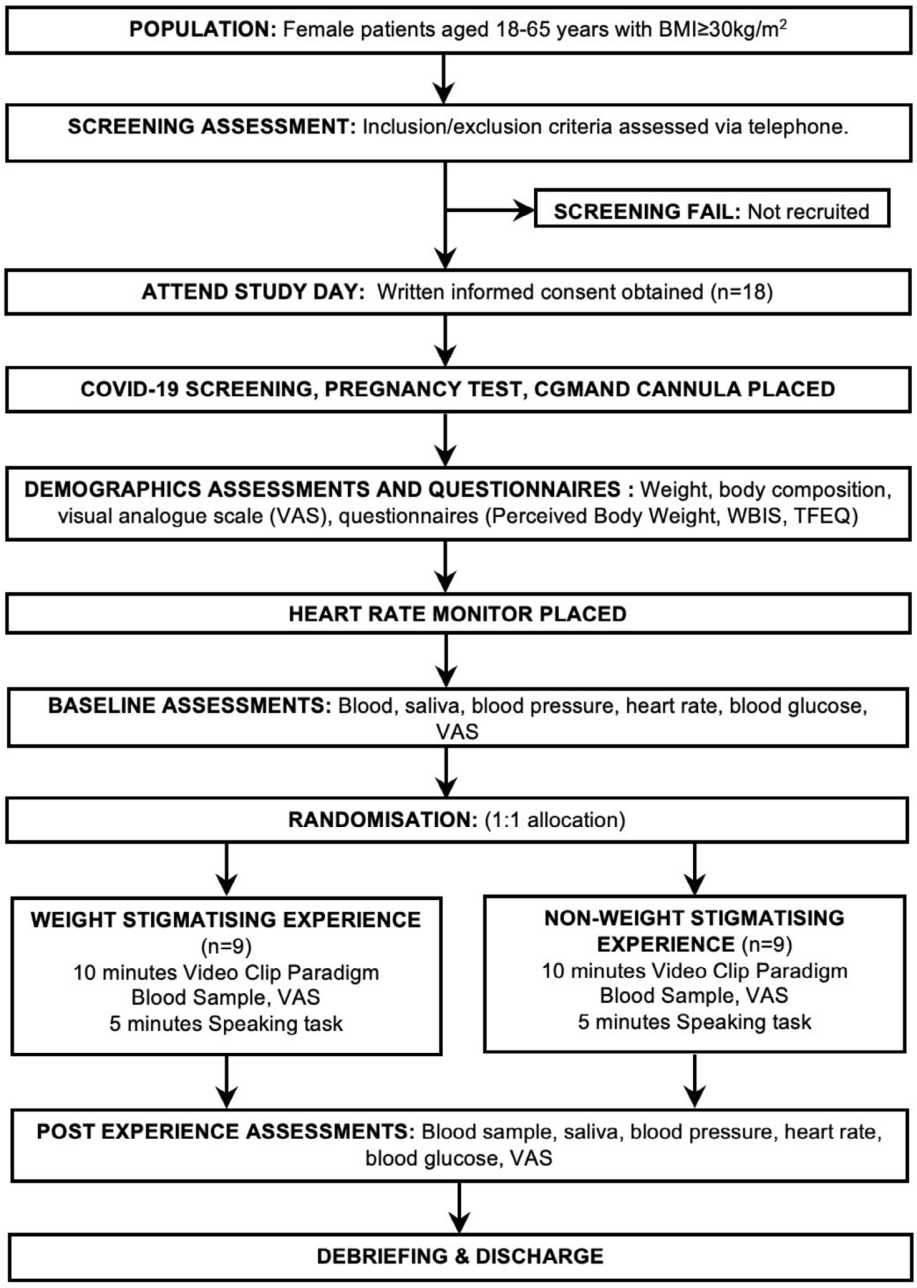
Participant flow diagram.

UK female adults living with obesity (body mass index [BMI] ≥ 30 kg/m^2^, aged 18–65 years) were invited to take part in the study. Potential participants were identified through the University College London Hospital Bariatric Centre for Weight Management and Metabolic Surgery and advertisement via social media, including Twitter and Facebook (Figure 1). Existing databases were searched for those meeting inclusion/exclusion criteria by a member of the clinical team (see Supporting Information for full inclusion/exclusion criteria). Potentially suitable participants were contacted via phone call by the clinical team to seek verbal consent to be approached by the research team with the aim of introducing the study, should they be interested. As this is an exploratory feasibility study and, at present, there was no data available looking at the physiological impact of weight stigma in patients living with obesity, a power calculation has not been done, and we aimed to recruit 20 participants, 10 in each group.

Eligible participants were contacted via phone call by the patient's clinical team to seek verbal consent to receive the PIS and gain verbal consent for the research team to contact them regarding a screening call. Members of the research team (A.B. and J.W.) also attended weekly bariatric education meetings to discuss the research with patients, and if patients were interested in participating in the study, their contact details were collected by the clinical team at the end of the session and were subsequently shared with the research team. Afterwards, the research team sent a PIS via post and/or email to those who expressed interest in participating. A convenient time was then arranged to discuss the study details and conduct the virtual screening (Figure 1).

At the virtual screening appointment, the research team explained the modified aims of the study, study day and answered any questions. To ascertain underlying chronic elevated perceived stress, the Perceived Stress Scale [27] was completed, with those having a score equal to or greater than 20 excluded. Those that were eligible were invited to the study visit. The first day of the last menstrual period in pre‐menopausal females was attained during this call. For pre‐menopausal women, an appointed study day during the luteal phase was identified to minimise the impact of cortisol response variation [28, 29]. For greater details on the study procedures, please see Supporting Information including the creation of the weight stigmatising and control experiences.

### 2.4 Study Day

Prior to attending the study visit (Figure S1), eligible individuals were asked to avoid caffeine, alcohol, and physical activity within 24 h of the assessment [30]. They ate a standardised breakfast, a minimum of 2 h prior to the scheduled visit. They attended the UCLH clinical research facility in the morning, where informed written consent was taken, after which a pregnancy and coronavirus (COVID‐19) screening test were conducted. If both were negative, participants continued with the visit. A cannula, a continuous glucose monitor (CGM, Dexcom G6, Dexcom Inc.) and a heart rate monitor (Polar M200, Polar, UK) watch and heart rate sensor (Polar H10, Polar, UK) were worn and the participant rested for approximately 2 h to allow for acclimatisation and calibration of the CGM. During this time anthropometric data were collected including weight, body composition, alongside three questionnaires: The Three Factor Eating questionnaire (TFEQ) [31], perception of own body weight [9] and Weight Bias Internalisation Scale (WBIS) [32]. Two hours prior to commencing the study, a visual analogue scale (VAS) was completed assessing self‐reported appetite and stress, followed by a standardised snack to avoid participants becoming excessively hungry prior to the experience, at which point another VAS was completed (See Supporting Information for VAS questions). Participants were then randomised to either a weight stigmatising or non‐weight stigmatising experience, of which they were blinded. Participants watched 10 min of media content that was either weight‐stigmatising or non‐stigmatising. They then completed a speaking task: those in the weight‐stigmatising group described a time they experienced weight stigma, while those in the non‐stigmatising group described their home (see Supporting Information for details). Following the rest period and between the hours of 1–2 pm, this was to address the diurnal pattern of cortisol [33], baseline measurements were taken for BP, venous bloods, saliva samples (Salivette Cortisol, Sarstedt) and another VAS was completed (t−15, t0 min). Both the weight stigmatising and non‐stigmatising paradigms consisted of two components, video clips and a speaking task.

At time point t0, participants randomised to the weight stigmatising experience were exposed to the initial 10 min video section of the weight stigmatising paradigm. Following a blood sample was taken (t10 min) and participants were asked to complete another VAS. Participants were then given written instructions regarding the speak aloud task of the weight stigmatising paradigm. Before starting, they were given a short period of thinking time. During both the passive video clip viewing and the speaking task the researchers were not present in the room, before returning to collect samples to allow the participants to focus on the task. At the end of the speaking task, further measurements were taken (t20: BP, saliva, blood and VAS). For the non‐stigmatising experience, the methodology was identical apart from the content of the paradigm described above. Further serial blood samples, BP and VAS were then taken following the end of the experiences 30, 45, 60, 90 and 120 min post completion of the weight stigmatising or non‐stigmatising experiences. Saliva samples were taken at 30, 45 and 120 min. In total, the visit duration was approximately 6 h. At the end of the study visit, participants received a full debrief and were provided with details of the true aims of the study. The debrief aimed to minimise any potential distress the participant might have felt from the experience.

### 2.5 Assays

Blood was collected as previously described [34, 35] Plasma cortisol, alpha amylase (AA), acyl‐ghrelin (AG), and PYY3‐36 were measured by ELISA. Insulin and vascular endothelial growth factor (VEGF) were measured using Luminex multiplex assays. ELISA were purchased from Tecan, Millipore, IBL International, Bertin Bioreagent and DLD Diagnostika GmbH and the Luminex multiplex assays from bio‐techne R&D systems.

### 2.6 Randomisation

Randomisation was conducted on the morning of the study day using a computerised 3rd party randomisation service (Sealed Envelope). Participants were randomised equally to either the weight stigmatising (experimental) or non‐weight stigmatising (control) experience at a 1:1 ratio controlling for BMI.

### 2.7 Data Analysis

The mean difference in cortisol at the end of the study day, between the weight stigmatising and non‐weight stigmatising experiences, was analysed using analysis of variance (ANOVA), adjusting for BMI and any baseline variables which are not balanced between the groups. Mean difference in cortisol was reported with 95% confidence interval and effect size. All available data were analysed. The results of the secondary analysis were treated as exploratory. Continuous outcomes were analysed using ANOVA, adjusting for BMI and any baseline variables which are not balanced between the groups. Time course data were analysed using two‐way mixed ANOVA. Mean differences in each outcome were reported with 95% confidence intervals and effect size. The assumptions of each model were checked and assumptions were met; if not, appropriate adjustments were made. If Mauchly's test of sphericity was not met, the Greenhouse–Geisser correction was used. *p* values were not reported as feasibility trials are not powered for testing hypotheses about effectiveness; rather to generate estimates of variability for the outcome measures and preliminary estimates of effect of the intervention [36].

For results from cortisol, inflammatory markers and gut hormone, the total and incremental area under the curve (AUC and iAUC, respectively) were calculated versus time using the trapezoidal rule. VAS responses were converted to a numerical value, and change over time was analysed by calculating the difference to baseline. Bias due to missing data was investigated and dealt with in an appropriate way for sensitivity analysis. A composite appetite score (CAS) was calculated from the VAS using the following formula in a way to more easily represent the data collected [37]: Appetite score = [Hunger + (100‐Fullness) + Desire to Eat + Appetite for Meal]/4. It is accepted that a mean score composed of key appetite scales can be used [38].

## 3 Results

### 3.1 Feasibility Outcomes

We successfully recruited 18 participants, achieving 90% of the recruitment target. Every participant completed the study protocol (100%), and no issues were reported regarding procedural burden or participant experience. The intervention was delivered as intended, with only minor operational considerations identified, specifically, ensuring timely placement of the CGM and cannula upon arrival to allow adequate calibration and acclimatisation time. Initially, two researchers (A.B. and J.W.) were required to collect samples at scheduled time points; however, with experience, this process could be managed by one researcher, with the other supporting the blood sample processing. No safety concerns arose during the study. Initial concerns were raised about potential distress related to the weight‐stigmatising experience; therefore, provisions were made for psychological support via a clinical psychologist. No participants accessed this support, and post‐study debriefs revealed no reports of distress. During the debrief session when revealing the purpose of the study, two participants within the weight stigmatising experience reported assuming that the study was related to weight stigma.

### 3.2 Participant Characteristics

Between 20th April and 6th September 2022, 18 female participants were recruited to the study (Figure 1), with a mean age of 43.2 years (SD 10.3), BMI of 45.8 kg/m^2^ (SD 5.9), SBP of 134.2 mmHg (SD 19.0), DBP of 73.2 mmHg (SD 12.1) and nearly three quarters White ethnicity (72.2% [*n* = 13]; Table 1). Participants had a mean WBIS score of 4.9 (SD 0.6) indicating high levels of internalised stigma, high levels of cognitive restraint (2.6 [SD 0.8]) and a perceived stress scale of 13.4 (SD 3.6; Table 1). In addition, participants perceived their own body weight to be either moderately heavy (16.7% [*n* = 3]) or very heavy (83.3% [*n* = 15]; Table 1). No differences were found between the intervention and control groups.

**TABLE 1:** Participant characteristics.

Characteristics, *n*	All participants	Weight stigmatising	Non‐weight stigmatising
Women, *n* (%)	18 (100)	9 (100)	9 (100)
Age, years (SD) (*n* = 600)	43.2 (10.3)	44.6 (9.0)	42.0 (11.9)
Ethnicity, *n* (%) (*n* = 645)			
Black African	2 (11.1)	1 (11.1)	1 (11.1)
Black Caribbean	1 (5.6)	0	1 (11.1)
Chinese	0	0	0
Indian	0	0	0
Middle Eastern	0	0	0
Mixed	2 (11.1)	1 (11.1)	1 (11.1)
Other	0	0	0
Pakistani	0	0	0
White	13 (72.2)	7 (77.8)	6 (66.7)
Bodyweight, kg (SD)	127.2 (18.6)	129.5 (16.1)	125.0 (21.6)
BMI, kg/m^2^ (SD)	45.8 (5.9)	48.2 (5.2)	43.4 (5.9)
Systolic blood pressure, mm Hg (SD)	134.1 (19.0)	139.4 (24.6)	128.8 (10.1)
Diastolic blood pressure, mm Hg (SD)	73.2 (12.1)	75.6 (14.0)	70.9 (10.2)
Hypertension, *n* (%)	1 (5.6)	1 (5.6)	0
Family history of obesity			
Father	6 (33.3)	4 (44.4)	2 (22.2)
Mother	10 (55.6)	6 (66.7)	4 (44.4)
Siblings	9 (50)	5 (55.6)	4 (44.4)
Questionnaires			
WBIS	4.9 (0.6)	5.1 (0.6)	4.7 (0.4)
TFEQ restraint	2.6 (0.8)	2.6 (1.0)	2.6 (0.6)
Perceived stress scale	13.4 (3.6)	13.1 (3.8)	13.7 (3.5)
Perceived body weight			
Very thin	0	0	0
Moderately thin	0	0	0
Slightly thin	0	0	0
Average	0	0	0
Slightly heavy	0	0	0
Moderately heavy	3 (16.7)	1 (11.1)	2 (22.2)
Very heavy	15 (83.3)	8 (88.9)	7 (77.8)

*Note:* Data are in *n* (%), mean (SD: standard deviation); median (IQR: interquartile range); %, percentage; *n*, number; BMI, body mass index, kg/m^2^, kilograms per metre squared, kg, kilograms; mmHg, millimetres of mercury; WBIS, weight bias internalisation scale; TFEQ, three factor eating questionnaire; no difference between group was found.

### 3.3 Preliminary Signals of Efficacy

Consistent with the study design, statistical significance was not sought; instead, the findings are preliminary signals that exposure to weight stigma experience may impact physiological response.

Key findings, below, from the study indicate that exposure to a weight stigmatising experience resulted in observed corresponding change in plasma cortisol, BP, self‐reported stress, appetite (hunger, fullness, desire to eat), and peptide YY. For detailed overview of results of other secondary outcome measures (AA, VEGF, AG, Glucose Insulin, anxiety and appetite) please see Supporting Information and (Figures S1–S3).

Time course data showed that following exposure to the weight stigmatising experience that there was an observed increase in plasma cortisol, SBP, self‐reported stress commencing at 10 min. Cortisol, self‐reported stress and hunger remained elevated until 30 min, while SBP peaked at 30 min. PYY however declined from 10 min to a nadir at 30 min, which coordinated with the decline in fullness from 20 min and greater desire to eat from 45 min.

### 3.4 Cortisol

The mean baseline cortisol for all participants was 58.9 (SD 22.6); mean cortisol of the experimental trial was 57.9 ng/mL (SD 23.0) and 59.9 ng/mL (SD 23.5) in the control trial (*p* = 0.856). There were no between group differences in cortisol at the end of the study day (21.5 ng/mL, 95% CI −34.7 to 77.7; partial *η* ^2^ = 0.06).

**FIGURE 2:**
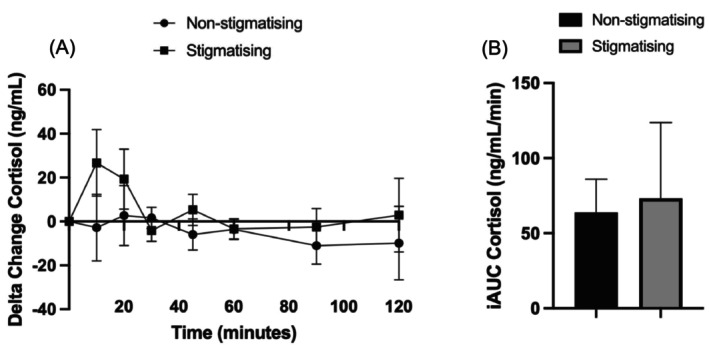
Comparison of inflammatory markers between weight stigmatising and non‐weight stigmatising groups over the time course of the paradigm and incremental area under the curve. (A) Time course data over the 120 min paradigm for serum cortisol; (B) iAUC_0–120_ for serum cortisol; iAUC, incremental area under the curve; ng/mL nanograms per millilitre; ng/mL/min, nanograms per millilitre per minute.

Time course data also showed there was no difference in plasma cortisol concentration between the intervention and the control (9.1 ng/mL, 95% CI −13.2 to 31.4, partial *η* ^2^ = 0.06; Figure 2a). Within‐group comparisons showed no change over time in cortisol compared to baseline for either group. However, an observed increase appeared 10 min following the weight stigmatising experience (26.7 ng/mL, 95% CI −5.5 to 58.9), which remained elevated until 30 min and then showed a sustained cortisol elevation until 120 min.

Cortisol levels analysed by AUC showed no difference between the intervention (8803.7 ng/mL [SD 6041.5]) compared with the control group (7673.0 ng/mL [2645.2], mean difference −1130.7 ng/mL, Cohen's *d* = −0.24, 95% CI −5791.1 to 3529.7). The iAUC_0–120_ for cortisol also showed no difference between the experimental and control trials (73.4 ng/mL [SD 50.3] vs. 63.9 ng/mL [SD 22.0] *d* = −0.24, 95% CI −48.3 to 29.4, respectively) (Figure 2b).

### 3.5 Blood Pressure

The mean baseline systolic blood pressure (SBP) for all participants was 134.1 mmHg (SD 19.0); mean SBP of the experimental trial was 139.4 mmHg (SD 24.6) and 128.8 mmHg (SD 10.1) in the control trial (*p* = 0.247). Diastolic blood pressure (DBP) for all participants was 73.2 mmHg (SD 12.1); 75.6 mmHg (SD 14.0) and 70.9 mmHg (SD 10.2) in the experimental and control trials, respectively.

**FIGURE 3:**
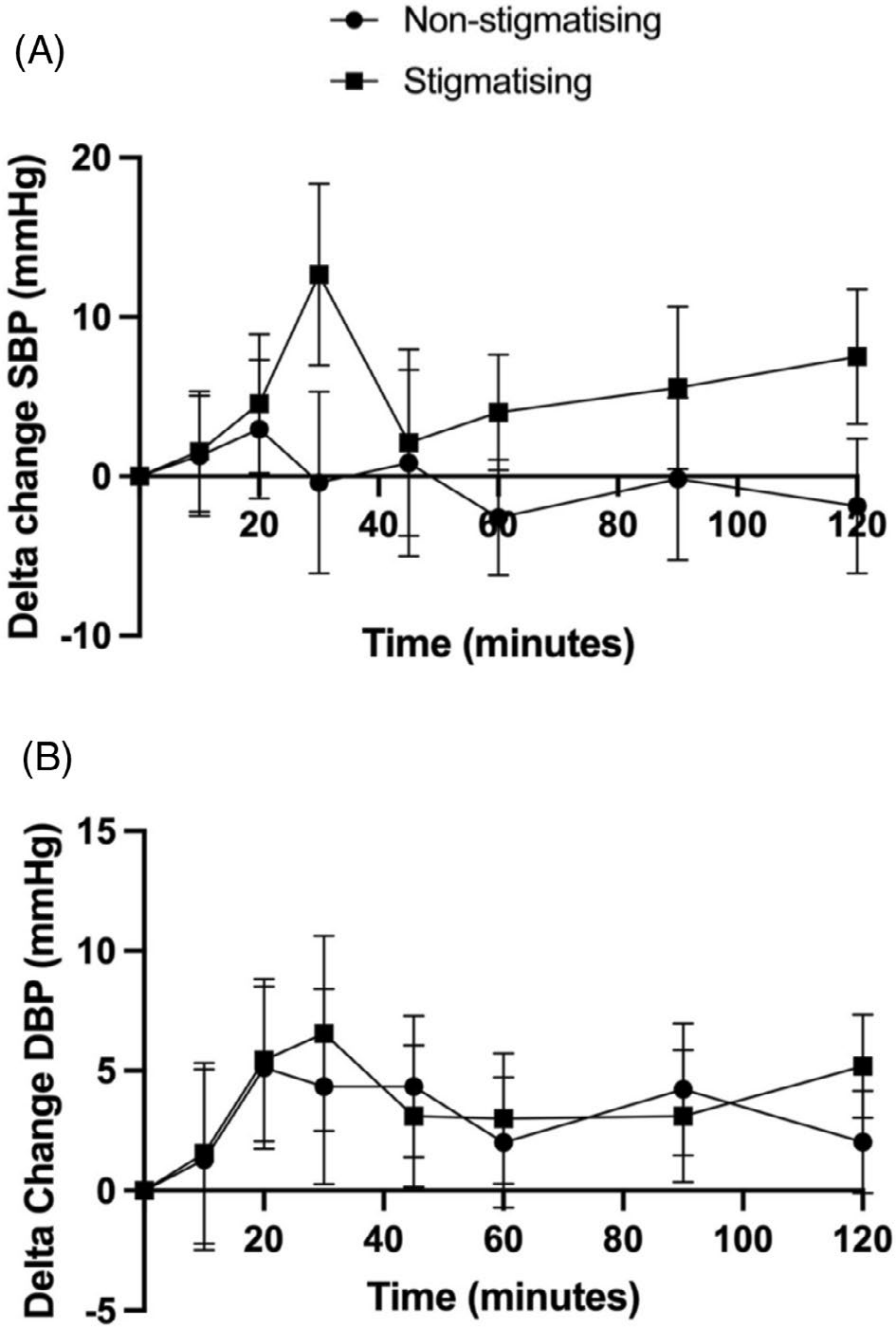
Comparison of systolic and diastolic blood pressure between weight stigmatising and non‐weight stigmatising groups over the time course of the paradigm. (A) Time course data over the 120 min paradigm for SBP; (B) Time course data over the 120 min paradigm for DBP. SBP, systolic blood pressure; DBP, diastolic blood pressure; mmHg, millimetres of mercury.

Time course data also showed that there was no difference in either SBP or DBP concentration between the intervention and the control (4.7 mmHg, 95% CI −4.2 to 13.6 partial *η* ^2^ = 0.05; 0.6 mmHg, 95% CI −5.5 to 6.7 partial *η* ^2^ = 0.01, Figure 3a,b, respectively). Within group comparisons showed no change in SBP compared to baseline for the control group; however, there was an observed increase compared to baseline between 10 and 30 min following the experimental trial and peaked at 30 min (weight stigma experience; 12.7 mmHg, 95% CI 0.6–24.8) (Figure 3b). Furthermore, SBP remained elevated compared to baseline throughout the study visit. Within group comparisons showed no change over time in DBP compared to baseline for either group apart from an increase at time point 120 min in the experimental group (5.2 mmHg, 95% CI 0.6–9.7) (Figure 3b).

### 3.6 Gut Hormones

#### 3.6.1 Peptide YY

The mean baseline Peptide YY (PYY) for all participants was 111.4 pg/mL (SD 63.4); mean PYY of 112.1 pg/mL (SD 65.9) and 110.8 pg/mL (SD 64.8) in the experimental and control trials, respectively.

**FIGURE 4:**
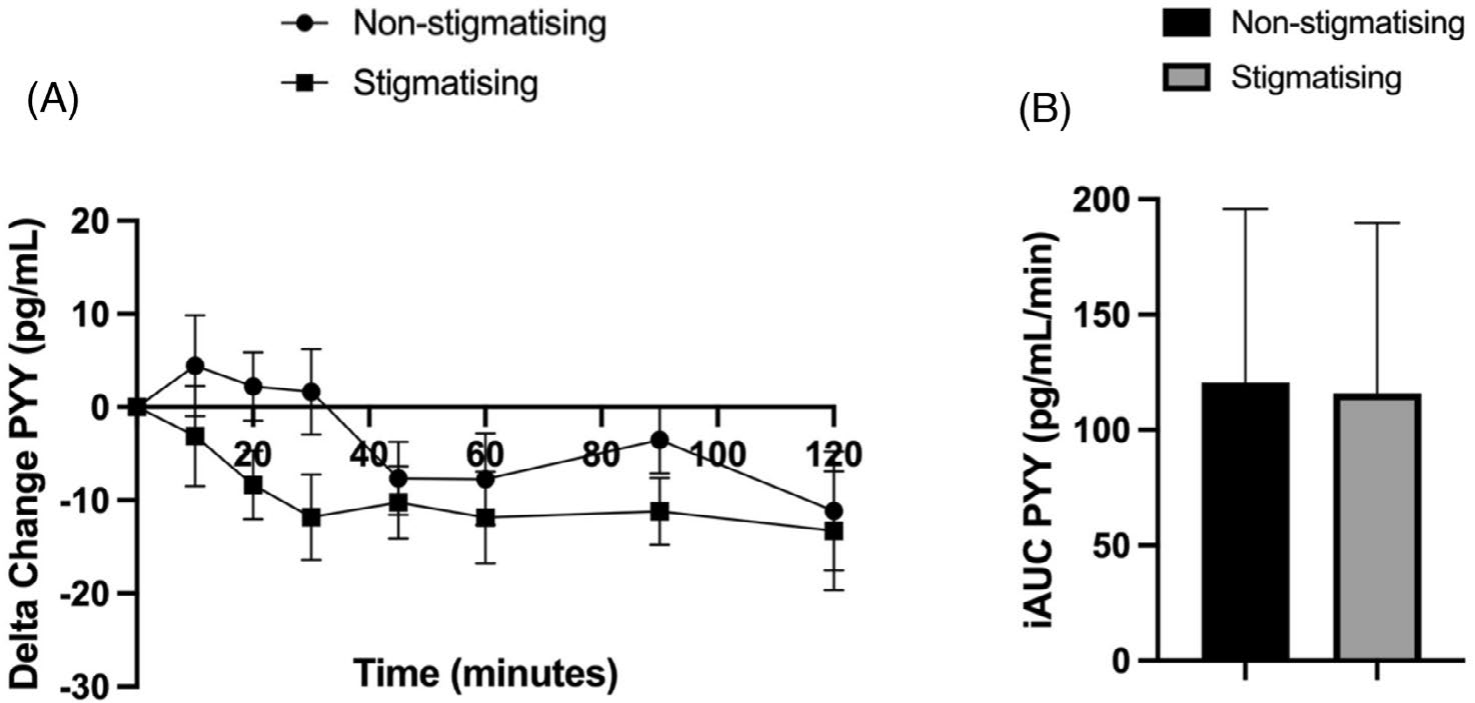
Comparison of PYY between weight stigmatising and non‐weight stigmatising groups over time course of the paradigm and incremental area under the curve. (A) Time course data over the 120 min paradigm for serum PYY; (B) iAUC_0–120_ for serum PYY. PYY, Peptide YY, iAUC, incremental area under the curve, pg/mL, picograms per millilitre, pg/mL/min, picograms per millilitre per minute.

Time course data also showed there was no difference in plasma PYY concentration between the experimental and control trials (−6.02 pg/mL, 95% CI −15.6 to 3.5, partial *η* ^2^ = 0.06; Figure 4a). Within‐group comparisons show no change over time in PYY compared to baseline for the control group. However, there was a reduction in PYY from 10 to 30 min following the weight stigmatising experience (−3.1 pg/mL, 95% CI −14.5 to 8.3; −8.4 pg/mL, 95% CI −16.1 to −0.6; −11.8 pg/mL, 95% CI −21.6 to −2.01, respectively), and then remained reduced from 45 to 90 min (−10.2 pg/mL, 95% CI −18.5 to −1.9; −11.9 pg/mL, 95% CI −22.4 to −1.4; −11.2 pg/mL, 95% CI −18.8 to −3.5, respectively).

PYY levels analysed by AUC showed no difference between the intervention (13907.3 pg/mL [SD 8858.5]) compared with the control group (14465.4 pg/mL [SD 9023.7], mean difference 558.1 pg/mL, Cohen's *d* = 0.062, 95% CI −8377.4 to 9493.6). The incremental iAUC_0–120_ for PYY also showed no difference between the intervention group compared to the control (115.9 pg/mL/min [SD 73.8] vs. 120.6 pg/mL/min [SD 75.2] *d* = 0.062, 95% CI −69.8 to 79.1) (Figure 4b).

### 3.7 Visual Analogue Scales

VASs were used to record subjective feeling on appetite throughout the study day. Both hunger and fullness were not different between the intervention and control (hunger; 37.0 mm [28.3]; 29.8 mm [27.2] 95% CI 13.1 to −34.9, fullness 49.3 mm [36.2]; 48.2 mm [38.6] 95% CI 17.6 to −38.5, respectively) before the standardised snack. Following the snack, a similar impact on both these parameters was observed across both groups with hunger reducing and fullness increasing (mean difference hunger −27.0 mm [21.1]; −18.3 mm [26.3]; mean difference fullness 22.8 mm [22.5]; 26.9 [27.6], respectively), showing the snack adequately suppress appetite prior to the start of the study protocol.

In addition, the mean baseline CAS was 42.5 (SD 27.7). There was no difference in mean CAS between the groups at baseline; mean CAS of 48.9 (SD 27.5) and 36.0 (SD 27.9; 95% CI −40.6 to 14.9) for the intervention and control, respectively or over time (6.9; 95% CI −9.5 to 23.2).

### 3.8 Stress

Over time, there was no difference in self‐reported stress between the intervention and the control (7.4 mm 95% CI −5.6 to 20.3, partial *η* ^2^ = 0.10). Within group comparisons showed no change in stress compared to baseline for the control group. However, there was an observed increase compared to baseline at 10 min following commencing the weight stigmatising experience (17.4 mm 95% CI 1.6–33.3) (Figure S4a) which was not observed in the control group.

### 3.9 Hunger

Over time, there was no difference in self‐reported hunger between the experimental and the control (8.3 mm 95% CI −8.5 to 24.7, partial *η* ^2^ = 0.01). Within group comparisons showed no change in hunger compared to baseline for the control group; however, there was an apparent observed increase in hunger starting at 10 min coordinating with exposure to the weight stigmatising experience, with an observed increase at 40 min (16.8 mm, 95% CI 2.0–31.7) and remaining elevated until 120 min (Figure S4b).

### 3.10 Fullness

Over time, there was no difference in self‐reported fullness between the intervention and the control (−5.9 mm 95% CI −18.6 to 6.8, partial *η* ^2^ = 0.05). Within group comparisons showed no change in fullness compared to baseline for the control group. However, there was an observed decrease compared to baseline at 40 and 120 min (−13.9 mm 95% CI −27.6 mm to −0.3; −18.3 mm 95% CI −35.9 to −0.7, respectively) (Figure S4c).

### 3.11 Desire to Eat

Over time, there was no difference in self‐reported desire to eat between the intervention and the control (9.9 mm 95% CI −7.5 to 27.4, partial *η* ^2^ = 0.02). Within‐group comparisons show no change in desire to eat compared to baseline for the control group. However, there was an observed increase compared to baseline at 45, 60, 90 and 120 min in the intervention group (20.9 mm 95% CI 5.1–36.7; 23.7 mm 95% CI 3.0–44.3; 24.8 mm 95% CI 2.5–47.1; 26.4 mm 95% CI 5.0–47.9, respectively) (Figure S4d).

## 4 Discussion

This is the first feasibility study to comprehensively measure the acute physiological impact of experiencing weight stigma in women living with obesity. Here we show that it is feasible to recruit, randomise and implement a novel weight stigma experience in female adults living with obesity. In addition we offer preliminary signals of efficacy, which expand on previous research [26, 34, 35], that suggest that experiencing weight stigma may have an observed impact not only on plasma cortisol but also on BP, self‐reported stress, appetite (hunger, fullness, desire to eat) and PYY. However, these findings need confirmation.

In addition, findings appear to show plasma cortisol, self‐reported stress, and SBP increase within 10 min of the weight‐stigmatising paradigm. This suggests that the weight stigmatising experience, using stigmatising videos and speaking task, successfully engendered a stress response related to weight stigma and met one of the initial objectives of the study.

The current study findings support previous work showing that experiencing weight stigma results in sustained cortisol elevation, where cortisol does not track the diurnal reduction in people living with obesity [9, 10, 11]. However, our findings extend those of earlier studies showing a rapid acute cortisol response (10 min) following weight stigma exposure. Salivary cortisol measurements have previously been taken 20–30 min following the stressor [9, 10, 11] due to delay in cortisol detection in saliva, whereas our frequent sampling revealed that cortisol appears to rise rapidly while experiencing weight stigma, enabling a more detailed characterisation of its response. This is of particular importance, as cortisol is involved in adipose tissue storage and distribution which are linked to the development of obesity‐related co‐morbidities [26]. Participants in our study perceived themselves to be either moderately or very heavy, which meant that comparing how perception of body weight impacted physiological response, which has previously been shown to affect cortisol response [9], was not possible.

Evidence has shown that, amongst people living with obesity, experiences of weight stigma impact eating behaviour including a greater likelihood of binge eating [39], comfort eating [1, 40], future weight gain [41] and increased calorie consumption [16, 42]. These previous study findings suggest that weight stigma experiences impact appetite regulation, though evidence explaining this is lacking.

Our findings suggest observed increases in self‐reported hunger and desire to eat following the weight stigmatising experience, with observed increases at 45 min and desire to eat remaining elevated until 120 min, though these changes were not statistically significant. Furthermore, there were observed reductions in self‐reported fullness between 45 and 90 min following the weight stigmatising experience.

When looking at plasma PYY, there was an observed acute reduction following the stigmatising experience, which remained below baseline between 10 and 90 min. This reduction may help explain the corresponding increases in hunger, desire to eat and reduced fullness, related to reduced satiety. PYY is a satiety hormone, with data showing that higher levels of PYY are related to reduced self‐reported appetite and increased food intake [43]. Acyl ghrelin, however, did not appear to change following response to weight stigma (Figure S3); therefore, this study offers a possible plausible biological mechanism to explain the previously observed changes in appetite following stigma [1, 16, 40, 42], in that they relate to a reduction in satiety instead of an increase in hunger. These responses in PYY and AG following stress have been reported elsewhere in women living with obesity [44, 45] though the impact on AG is inconsistent [46, 47]. In addition, previously, data have recognised cortisol as a mediator of stress‐induced eating, specifically a drive to eat foods high in fat and sugar [48, 49], suggesting an additional possible pathway related to the impacts on appetite. Further studies are needed to confirm this finding, though these data expand evidence to date.

Although no difference was found in BP between the groups, there was an observed increase in SBP during the weight stigmatising experience, peaking at 30 min with an increase of 12.7 mmHg, which then remained elevated compared to baseline throughout the remainder of the study visit. This acute response may have been through various mechanisms, some of which we measured. The BP reactivity corresponded with an acute response in cortisol described above, with cortisol being shown to interact with the sympathetic nervous system contributing to increased BP [50], this could partially explain this response. Previous data have also shown that, in response to experiences of other social stigmas such as racism, cortisol causes an increase in heart rate and BP [21, 22], impacting on inflammatory hormones [23, 24, 25]. Furthermore, we also tentatively suggest that BP changes observed may be due to changes in catecholamines, namely adrenaline, nor‐adrenaline and dopamine. However, due to the challenges analysing these data, we are unable to confirm this potential explanation. As such, future studies are needed that use high sensitivity assays to gain further understanding about the mechanism of this apparent acute physiological response.

This BP reactivity to weight stigma in a group with slightly elevated baseline BP is of concern, as short term BP variability has been associated with increased cardiovascular events, mortality and target‐organ damage [51, 52] Limited previous data exist on stress induced BP reactivity [15, 53] with recent experimental data showing elevation in systolic (~6 mmHg) and diastolic (~4 mmHg) BP immediately after acute exposure to weight stigma, which persisted until sleep hours using ambulatory monitor [53]. Within our study we did not show an increase in HR when continuously measured, though there was a slight observed increase in heart rate at 75 min within the intervention group, though this remained within normal physiological parameters for people living with obesity [54].

Previous data has shown that experiences of weight stigma are associated with elevated glycated haemoglobin in people without diabetes with higher central adiposity [55]. While those living with Type 2 diabetes experiencing stigma report worse diabetes self‐care, worse diabetes outcomes and elevated diabetes‐specific distress after accounting for depression, BMI and other confounders [56]. Thus, understanding how experiencing weight stigma on glycaemia, both response on plasma glucose and insulin, may help to further explore the mechanisms driving these findings. Despite these previous findings, there were no observed changes in glucose and insulin data (Figure S4a–d) between either group suggesting that experiencing a weight stigma experience does not acutely impact glycaemia as potentially expected. Despite this, the time course data appears to show observed differences to the expected postprandial response [57] in those experiencing weight stigma. Following meal ingestion, both plasma insulin and glucose increase, which is followed by a corresponding reduction in glucose and insulin back to baseline [57]. This normal physiological response can be seen following the non‐stigmatising experience, though experiencing weight stigma appears to result in apparent observed changes in insulin and glucose. The changes may be explained as a response to cortisol directly suppressing insulin release and insulin secretion [58], and stress caused a ‘flight or fight’ response, mobilising the body' energy stores, with a rapid release of endogenous glucose from the liver [59]. Unfortunately, despite collecting catecholamines, as explained previously data were not available, which could have helped to further explain this response. This possible alteration in glucose and insulin response following experiencing weight stigma may help to further explain the impact stigma has on glycaemia, though further studies are required to confirm these proposed mechanisms.

The current study created a novel weight stigmatising experience that permitted the measurement of real‐time physiological responses of multiple outcome measures. This study has shown recruitment, randomisation and measuring real‐time physiological response is feasible, and future, appropriately powered research will hopefully provide more conclusive evidence on the acute physiological impact of weight stigma. This feasibility study recruited only a small sample of 18 participants, all being female, and therefore was not sufficiently powered to observe significant changes in the key outcomes and cannot be generalised. This may help to also explain the lack of observed changes in other secondary markers including AA, VEGF and strength of appetite. This means there remains to be limited convincing evidence that stigma acutely impacts physiology compared to non‐stigmatising experiences. Future research should also aim to look at the physiological impact of weight stigma on different genders, ethnicities and BMI ranges to gain greater understanding and generalisability. Despite this, the study does indicate that experiencing weight stigma appears to have an observed physiological response. Another limitation was that AG was the only measured form of ghrelin, which might not explain the full picture related to ghrelin response and therefore future studies should aim to measure deacyl‐ghrelin as well. In addition, catecholamine levels were unexpectedly low, preventing interpolation and thus reporting concentrations of adrenaline, noradrenaline, and dopamine. These data could have clarified BP, glucose, and insulin changes linked to the fight‐or‐flight response; therefore, future studies should use high‐sensitivity assays to measure these markers.

## 5 Conclusion

The current feasibility study shows that it is feasible to recruit, randomise and measure the real‐time physiological response of multiple physiological outcomes to a novel weight stigmatising experience. These data offer possible mechanistic explanations for the observational data regarding the negative impact of weight stigma on physiological health on appetite and BP and stimulate new avenues of investigation for future studies.

## Author Contributions

A.B. and J.W. conceived the study. A.B., J.W. and S.W.F. contributed to the study design and methodology. A.B. was responsible for the oversight of the study. A.B. and J.W. contributed to the recruitment of participants. A.B., J.W. and S.W.F. were responsible for the data analysis. A.B. and J.W. authors contributed to initial data interpretation and the writing of the first draft manuscript. All authors contributed to critical revision of the manuscript and gave final approval.

## Funding

This research was supported by a competitive grant by Rosetrees Trust (UCL‐Obesity‐2020\102) and the Obesity Theme of the University College London/UCLH Comprehensive Biomedical Research Centre (BRC781/OB/AB/110370), which received a proportion of its funding from the Department of Health's NIHR Biomedical Research Centres.

## Conflicts of Interest

A.B. declares researcher‐led grants from the National Institute for Health Research, Rosetrees Trust, MRC, INNOVATE UK, British Dietetic Association, British Association of Parenteral and Enteral Nutrition, BBRSC, the Office of Health Improvement and Disparities and NovoNordisk. A.B. reports travel expenses for attending academic conferences from Eli Lilly. A.B. reports honoraria from Novo Nordisk, Eli Lilly, Nutricia and Mac Nutrition outside the submitted work and is on the Medical Advisory Board and shareholder of Reset Health Clinics Ltd. S.W.F. declares researcher‐led grants from the National Institute for Health Research, Office of Health Improvement and Disparities and Novo Nordisk and support for attending academic conferences and events from Novo Nordisk, Eli Lilly, UK Parliament, Welsh Parliament and Safefood. J.W. declares no conflicts of interest.

## Supporting information


**Data S1:** cob70073‐sup‐0001‐Supinfo.pdf.

## Data Availability

The data that support the findings of this study are available from the corresponding author upon reasonable request.
