# Supplementary material for: A Randomised Feasibility Study Assessing Acute Physiological Responses to Weight Stigma in Women Living With Obesity
Source: Clin Obes. 2026 Feb 15;16(2):e70073. doi: 10.1111/cob.70073 (PMC12906996; doi:10.1111/cob.70073)
Supplement: Supplementary file 1 — Data S1: cob70073‐sup‐0001‐Supinfo.pdf. [file COB-16-e70073-s001.pdf]

A randomised feasibility study assessing acute physiological responses to weight stigma in women living with obesity

Adrian Brown PhD,[1,2,3*] Jed Wingrove, PhD[1], Stuart W. Flint PhD [4,5]

1. Centre for Obesity Research, University College London, London, UK
2. Bariatric Centre for Weight Management and Metabolic Surgery, University College London Hospital NHS Trust, London, UK
3. National Institute of Health Research, UCLH Biomedical Research Centre, London, UK
4. School of Psychology, University of Leeds, Leeds, UK
5. Scaled Insights, Nexus, University of Leeds, Leeds, UK

Corresponding Author: Dr Adrian Brown, Centre for Obesity Research, University College London, London, UK; Email: a.c.brown@ucl.ac.uk Telephone: 02076790788 (*Senior Author)

Supplementary Material

Inclusion/Exclusion Criteria

Inclusion criteria

Patients will be considered for the study if they meet the following criteria:

1. Female adults aged 18-65 years.
2. Body Mass Index equal or greater than 30 kg/m^2.
3. Weight stable at time of recruitment, defined as less than 5% variation in body weight over the preceding 3 months.
4. Proficient in written and spoken English.
5. Able to comply with study protocol.
6. Willing and able to provide written informed consent.
7. Being registered with a GP and willingness for their GP to be informed of their participation in the study.
8. Able to access the internet.

Exclusion criteria

Patients will be excluded for any of the following reasons:

1. Type 1 and type 2 diabetes (in view of altered circulated gut hormone profiles and gustatory function).
2. Smoking (in view of the fact that smoking affects salivary cortisol).
3. Diagnosed with uncontrolled severe depression.
4. Diagnosed with uncontrolled psychiatric disorder.
5. Previous bariatric surgery.
6. Acute illness or chronic conditions that may impact HPA including Cushing syndrome.
7. Known or suspected history of HIV, Hepatitis B or C or other blood−borne diseases (in view of safety regulations regarding exposure to blood products).
8. Currently using glucocorticoids.
9. Pregnancy or lactation.
10. Elevated self-perceived stress as assessed by the Perceived Stress Scale.

Additional Methodology

CGM

Glucose variability was assessed using a Dexcom G6 (real-time continuous glucose sensor system; Dexcom, Inc. US). The Dexcom G6 system is a CE marked real-time continuous glucose sensor system, which provides dynamic glucose information. These glucose data were updated every 5 minutes. The participants wore the Dexcom G6 continuous glucose sensor in the anterior abdominal wall, where the sensor detects glucose in the interstitial fluid (reference).

Vital signs

Throughout the study visit heart rate was continuously measured using a Polar M200 watch and Polar H10 heart rate sensor (Polar, UK). Blood pressure was monitored using a standing blood pressure machine with appropriately sized cuff at the following time points, -15, 0, 20, 30, 45, 60, 90 and 120 minutes.

Blood and saliva samples

Throughout the study visit blood and saliva samples were taken. Blood samples were taken at the following time points, -15, 0, 10, 20, 30, 45, 60, 90 and 120 minutes. Saliva samples was also taken at -15, 20, 30, 45 and 120 minutes. Blood samples were collected through the cannula placed in the participant’s arm. Saliva samples were collected using a salivette (Salivette® Cortisol, Sarstedt) in order to measure salivary cortisol and α-amylase.

Example of Study Timeline

Day -1 Participants asked to avoid caffeine, alcohol and physical activity 24 hours prior to study day

Day 0 (STUDY DAY)

0900 Participants consume standardised breakfast at home
1000 Participants arrive at clinical facility and written consent is obtained
1020 COVID-19 screening and pregnancy test
1030 Cannula and CGM placed– 2 hour acclimatisation period before first sample/measure
1040 Anthropometric measurements recorded – Height, Weight, BIA, VAS
1050 Participants given a standardised snack, VAS
1100 Questionnaire completion
1300 Polar monitor, for continuous monitoring of heart rate & HR variability,
1330 T-15 Assessment – BP, blood, saliva, VAS
1345 T0 Assessment – BP, blood, VAS & start weight stigmatising/non-stigmatising experience
1355 T10 Assessment at end of Video Clip Paradigm – blood, VAS & Speaking Task commences
1400 T20 Assessment at end of Speaking Task – BP, blood, saliva, VAS
1415 T30 Assessment – BP, blood, saliva, VAS
1430 T45 Assessment – BP, blood, saliva, VAS
1445 T60 Assessment – BP, blood, VAS
1515 T90 Assessment – BP, blood, VAS
1545 T120 Assessment – BP, blood, saliva, VAS
1550 CGM, cannula and Polar monitor removed
1600 Debriefing
1630 Discharged

Figure S1: Outline study visit to determine the physiological response to a weight stigmatising experience.

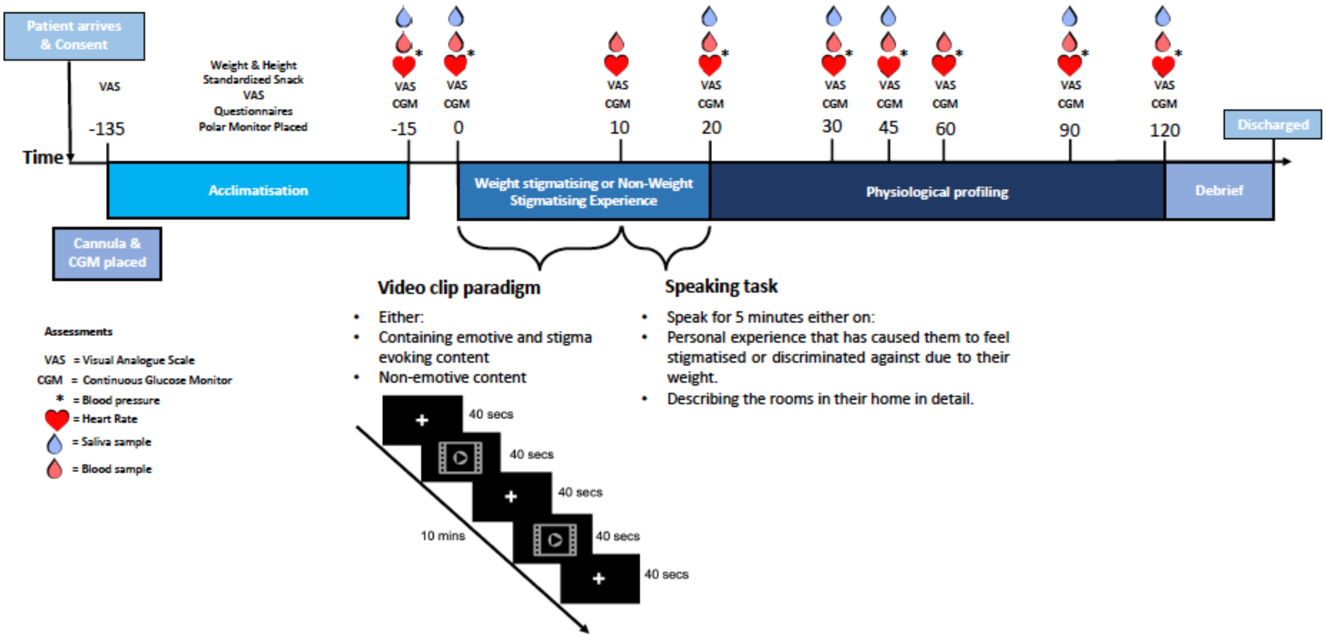

Creation of the weight stigmatising and control experiences

To explore the impact of weight stigma on people living with obesity, a novel weight stigmatising experience trial and a matched non-weight stigmatising control trial were developed. To achieve this and to ensure the weight stigma experience reflected typical experiences, people living with obesity were invited to participate in online focus groups. Focus groups were designed to discuss the previous research relating to weight stigmatising paradigms and different types of stigmatising media content containing brief clips of television or movie clips that depicted people living with obesity experiencing weight stigma (1). Beforehand, weight stigma experts from across the world were recruited and asked to assign each media clip into key themes (e.g., questioning weight stigma, blatant weight stigma discrimination and experience of weight stigma). Within this process, focus group participants rated each stigmatising clip media; ratings were used to select the most appropriate content for the weight stigma experience used in the experimental trial. This created the initial 10-minute section of the experimental trial. The non-weight stigmatising media content consisted of brief clips of non-weight stigmatising scenes which were unrelated to weight. Each clip was approximately 40-60 seconds in length and was followed by a blank screen period. These clips were matched to the weight stigmatising clips in terms of context and format i.e., video clip from same comedy show or television show one which depicted weight stigma and the other non-weight stigma. This aimed to create clips that were both visually and contextually similar, with only the content and message differing. This was to ensure any potential stress caused by the show, presenters or characters was controlled for.

The experimental paradigm was further developed through the use of a novel speaking task lasting five minutes. From research methodology used for expressive writing tasks related to stigma (a variation of the approach used by Pennebaker & Beall, 1986 (2), we created a novel speaking task, which either asked the participant to discuss in detail a stigmatising event that they had personally experienced. They were asked to provide as much information as they could and try to convey how they felt during that particular event. This in combination with the weight stigmatising experience (media content), formed the novel weight stigmatising experience experimental trial. The control arm of the speaking trial required individuals to undergo a speaking task albeit they were asked to describe in detail the rooms in their home. In total the paradigm aimed to last a total of 15 minutes.

Secondary outcome results

Inflammatory Markers

Alpha-amylase

The mean baseline alpha-amylase (AA) for all participants was 196.3 U/mL (SD 139.8) with those in the intervention have an AA of 206.3 U/mL (SD 145.3) and in the control 186.4 U/mL (SD 142.1).

Figure S2a shows the delta change in AA over serial measurements of the weight stigma experience. Time course data also showed no difference in plasma cortisol concentration between the experimental and the control trials (-0.14 U/mL, 95% CI -63.5 to 63.2 partial η^2=0.04). Within group comparisons showed no change over time in AA compared to baseline for either group. However, AA increased at 45 minutes within the intervention group.

AA levels analysed by area under the curve (AUC) showed no difference between the intervention (8181.6 U/mL [SD 4592.1]) compared with the control group (8141.0 U/mL [SD 4881.2], mean difference -40.6, Cohen's d=-0.09, CI 95% -4776.3 to 4965.2). The incremental $iAUC_{0-120}$ for AA also showed no difference between the experimental compared to the control group (68.2 U/mL/min [SD 38.2] vs 67.8 U/mL/min [SD 40.7] Cohen's d=-0.09, CI 95% -39.8 to 39.1) (Figure S2b).

Vascular Endothelial Growth Factor

The mean baseline VEGF for all participants was 17.7 ng/ml (SD 13.1); mean VEGF of 19.1 ng/mL (SD 15.2) and 15.8 ng/mL (SD 10.7) for the experimental and control trials respectively.

Figure S2c shows the delta change in VEGF over serial measurements of the weight stigma paradigm. Time course data also showed there was no difference in plasma VEGF concentration between the intervention and the control (-3.10 ng/mL, 95% CI -8.53 to 2.35, partial η^2=0.11). Within group comparisons showed an apparent reduction in VEGF at 45 minutes in the control group and within the intervention group a reduction between 45-90 minutes.

VEGF levels analysed by area under the curve (AUC) showed no difference between the experimental (2126.9 ng/mL [SD 1758.7]) compared to the control group (4138.7 ng/mL [SD 8137.3], mean difference 2011.8, Cohen's d=0.342, CI 95% -3871.1 to 7894.7). The incremental $iAUC_{0-120}$ for VEGF also showed no difference between the intervention group compared to the control (17.7 ng/mL/min [SD 14.7] vs 34.5 ng/mL/min [SD 67.8] d=0.342, CI 95% -32.3 to 65.8) (Figure S2d).

Acyl ghrelin

The mean baseline AG for all participants was 31.9 pg/mL (SD 31.2) with those in the intervention having an AG of 26.2 pg/mL (SD 29.0) and in the control 37.7 pg/mL (SD 34.1).

Figure S3a shows the delta change in AG over serial measurements of the weight stigma paradigm. Time course data also showed there was no difference in plasma AG concentration between the intervention and the control (0.71, 95% CI -7.19 to 8.60, partial η^2=0.12), though within group comparisons showed reductions over time in AG within both groups.

AG levels analysed by AUC showed no difference between the intervention (3232.3 [SD 3652.9]) compared with the control group (4515.2 pg/mL [SD 4643.5], mean difference 1282.9 pg/mL, Cohen's d=0.307, CI 95% -2892.0 to 5457.8). The $iAUC_{0-120}$ for AG also showed no difference between the intervention compared to the control group (26.9 pg/mL/min [SD 30.4] vs 37.6 pg/mL/min [SD 38.7] Cohen's d=0.307, CI 95% -24.1 to 45.5) (Figure S3b).

Glucose and insulin

Insulin

The mean baseline insulin for all participants was 839.5 pmol/L (SD 781.5); mean insulin of 792.8 pmol/L (SD 710.2) and 870.7 pmol/L (SD 866.5) in the experimental and control trials respectively.

Figure S4a shows the delta change in insulin over serial measurements of the weight stigma experience. Time course data also showed there was no difference in plasma insulin concentration between the experimental and control trials (-104.8 pmol/L, 95% CI -470.8 to 261.30, partial η^2=0.05). Within group comparisons showed no change over time in insulin compared to baseline within the control group, though within the weight stigmatising group there was an acute reduction at 20 minutes in insulin that remained lower than baseline throughout the study period, in addition there was a reduction at 90 minutes.

Insulin levels analysed by AUC showed no difference between the intervention (95019.4 pmol/L [SD 82584.7]) compared with the control group (74690.4 pmol/L [SD 70190.6], mean difference -20239.1 pmol/L, Cohen's d=-0.265, CI 95% -96916.5 to 56258.5). The $iAUC_{0-120}$ for insulin also showed no difference between the experimental compared to the control group (791.8 pmol/L/min [SD 688.2] vs 622.4 pmol/L/min [SD 584.9] d=-0.265, CI 95% -807.6 to 468.8) (Figure S4b).

Blood glucose

The mean baseline blood glucose for all participants was 5.3mmol/L (SD 1.2); mean blood glucose of 5.4 (SD 0.90) and 5.3 (SD 1.5) in the experimental and control trials respectively.

Figure S4c shows the delta change in blood glucose over serial measurements of the weight stigmatising experience. Time course data also showed there was no difference in blood glucose concentration between the intervention and the control (-.062, 95% CI -0.51 to 0.39, partial η^2=0.07). Within group comparisons showed no change over time in blood glucose compared to baseline in the control group. However, there was an observed reduction in blood glucose compared to baseline at between 10 to 35 minutes following the weight stigmatising experience. The peak reduction was at 20 minutes (-0.82, 95% CI -1.3 to -0.34) which corresponding with the weight stigmatising paradigm finishing.

Glucose levels analysed by AUC showed no difference between the experimental (92.5 mmol/L [SD 46.5]) compared with the control group (67.5 mmol/L [SD 36.1], mean difference -25.0, Cohen's d=-0.606, CI 95% -67.8 to 17.7). The $iAUC_{0-120}$ for glucose also showed no difference between the intervention group compared to the control (0.68 mmol/L [SD 0.45] vs 0.56 mmol/L [SD 0.30] d=-0.325, CI 95% -0.50 to 0.26) (Figure S4d).

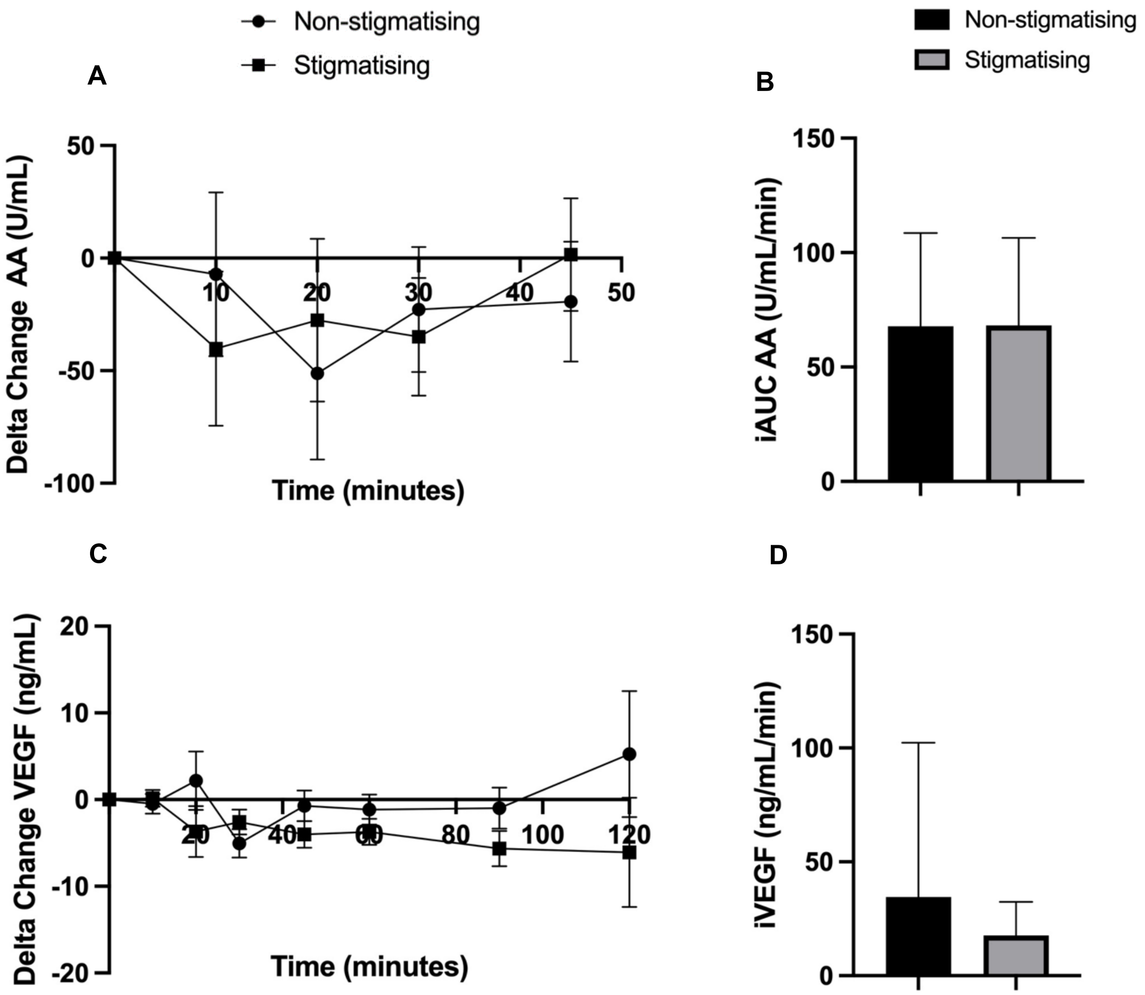

Figure S2 Comparison of inflammatory markers between weight stigmatising and non-weight stigmatising groups over time course of the paradigm and incremental area under the curve. S1a, Time course data over the 120-minute paradigm for salivary AA; S1b, $iAUC_{0-120}$ for salivary AA; S2c, Time course data over the 120-minute paradigm for serum VEGF S2d, $iAUC_{0-120}$ for serum VEGF

AA, salivary alpha amylase, VEGF, Vascular Endothelial Growth Factor, iAUC, incremental area under the curve, U/mL, units per millilitre, ng/mL nanograms per millilitre, U/mL/min, units per millilitre per minute, ng/mL/min, nanograms per millilitre per minute

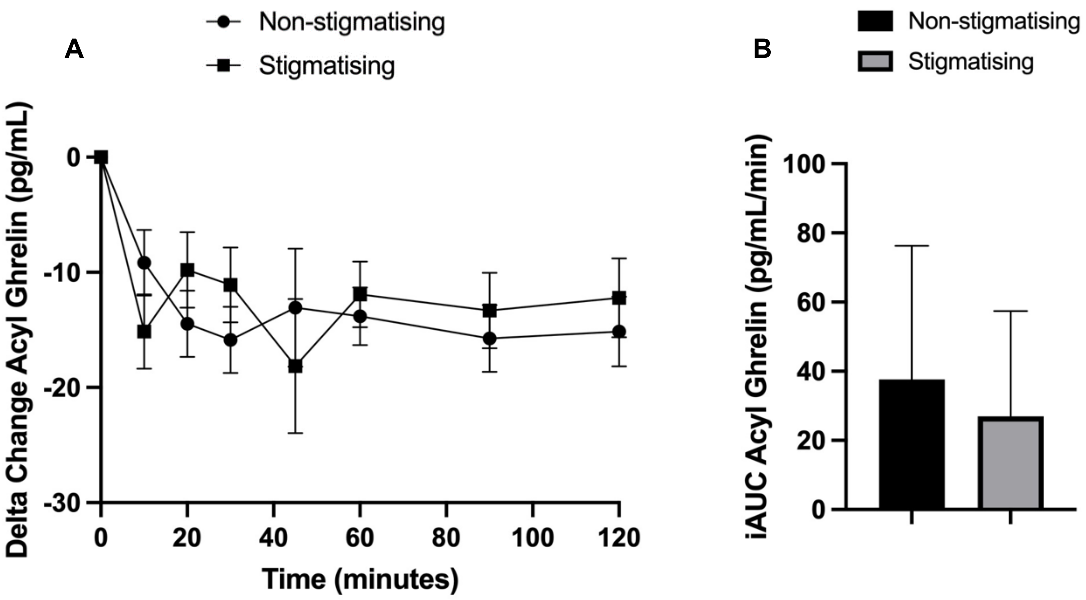

Figure S3 Comparison of Acyl-Ghrelin between weight stigmatising and non-weight stigmatising groups over time course of the paradigm and incremental area under the curve S2a, Time course data over the 120 minute paradigm for serum acyl ghrelin; S2b, $iAUC_{0-120}$ for serum acyl ghrelin.

iAUC, incremental area under the curve, pg/mL, picograms per millilitre, pg/mL/min, picograms per millilitre per minute

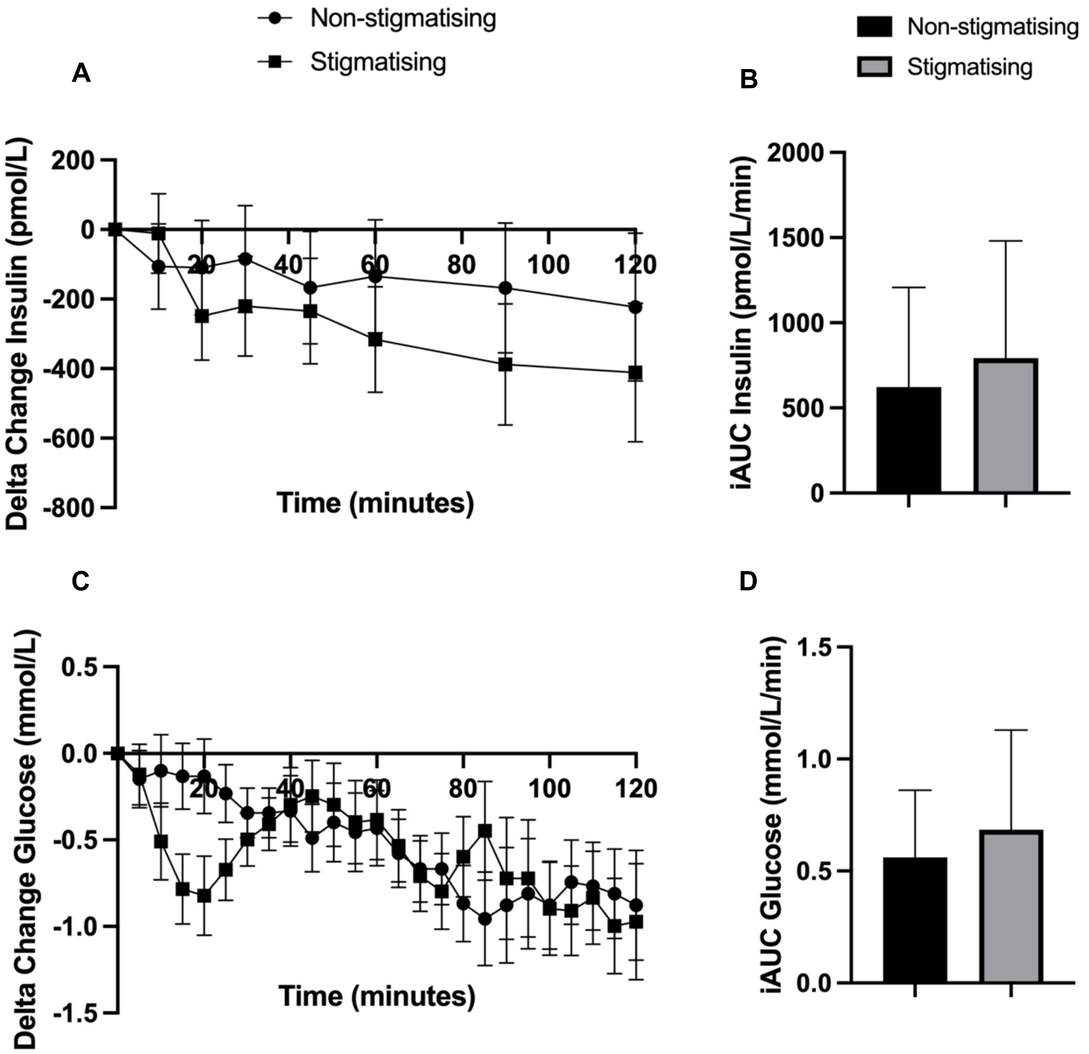

Figure S4 Comparison of glucose and insulin between weight stigmatising and non-weight stigmatising groups over time course of the paradigm and incremental area under the curve S3a, Time course data over the 120 minute paradigm for serum insulin; S3b, iAUC$_{0\text{-}120}$ for serum insulin; S3c, Time course data over the 120 minute paradigm for glucose; S3d, iAUC$_{0\text{-}120}$ for glucose.

iAUC, incremental area under the curve, pmol/L, picomole per litre, pgmol/L/min, picomole per litre per minute; mmol/L, millimole per litre; mmol/L/min, millimole per litre per minute

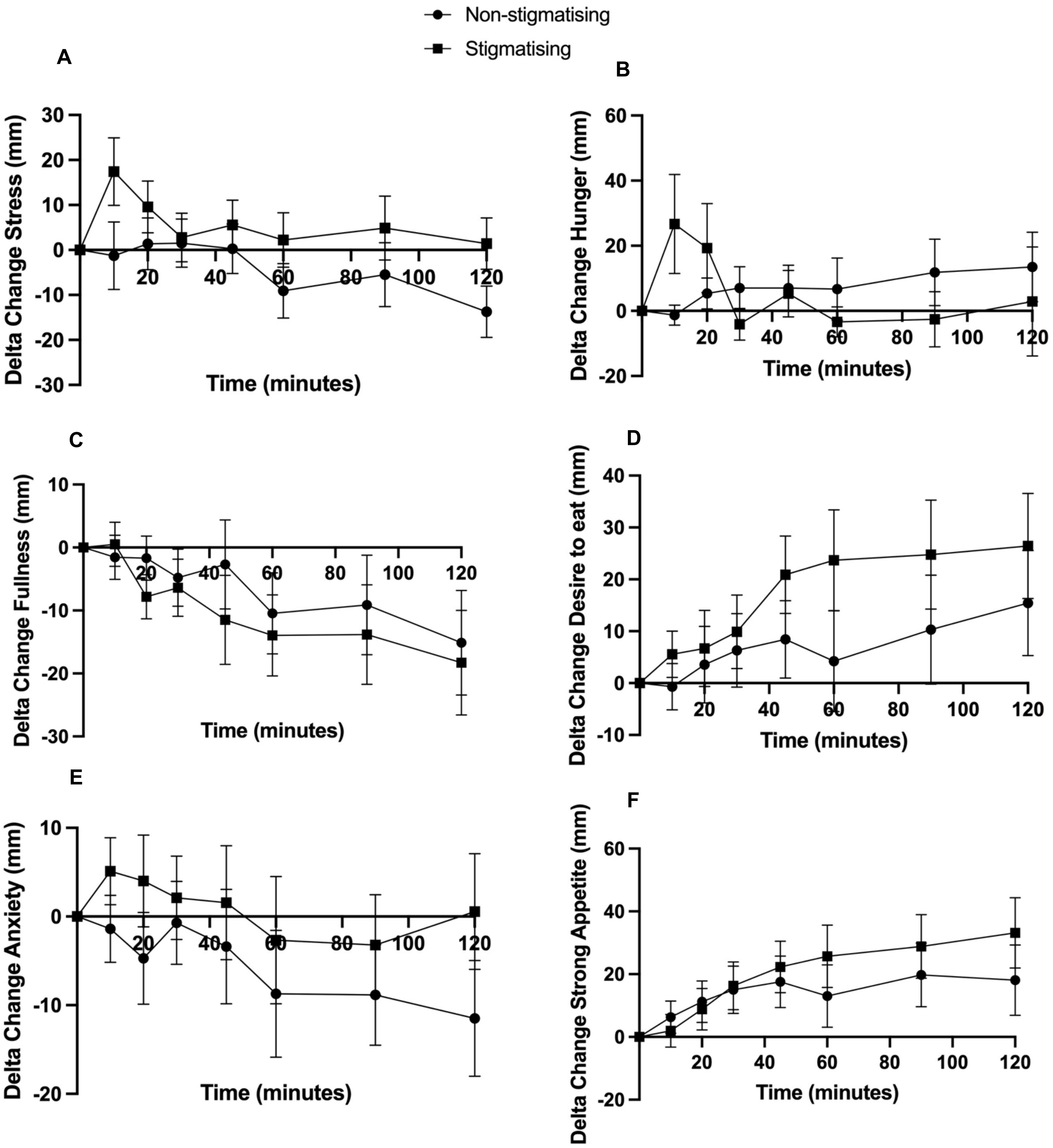

Figure S5 Comparison of visual analogue scales between weight stigmatising and non-weight stigmatising groups over time course of the paradigm and incremental area under the curve. S4a, Time course data over the 120 minute paradigm for stress VAS; S4b Time course data over the 120 minute paradigm for hunger; S4c Time course data over the 120 minute paradigm for fullness; S4d Time course data over the 120 minute paradigm for how strong is your desire to eat. S4e Time course data over the 120 minute paradigm for anxiety; S4f Time course data over the 120 minute paradigm for how strong is your appetite;

mm, millimetres

References

1. Schvey NA, Puhl RM, Brownell KD. The Stress of Stigma: Exploring the Effect of Weight Stigma on Cortisol Reactivity. Psychosomatic medicine. 2014;76(2).

2. Pennebaker JW, Beall SK. Confronting a traumatic event: Toward an understanding of inhibition and disease. Journal of Abnormal Psychology. 1986;95(3):274-81.
